# Toxicological evaluation of aqueous leaf extract of *Terminalia catappa* linn (combretaceae) in pregnant rats

**DOI:** 10.11604/pamj.2023.46.118.37339

**Published:** 2023-12-28

**Authors:** Murtala Abdullahi, Shehu Yakubu Magaji, Zainab Gambo Ibrahim, Suleiman Yunusa, Agbo John, Ibrahim Muhammad, Yusuf Abubakar Muhammad, Sani Malami, Basheer Zubairu Chedi

**Affiliations:** 1 Department of Pharmacology and Therapeutics, Faculty of Pharmaceutical Sciences, Bayero University Kano, Kano, Nigeria,; 2 Department of Clinical Pharmacology and Therapeutics, College of Medical Sciences, Abubakar Tafawa Balewa University, Bauchi, Nigeria,; 3 Department of Pharmacology, Faculty of Basic Medical Sciences, Bauchi State University Gadau, Bauchi, Nigeria,; 4 Venom-Antivenom Research Project (VASP) and Nigeria- Snakebite Research and Intervention Centre (N- SRIC), Kano, Nigeria

**Keywords:** Toxicity, pregnancy, *Terminalia catappa* linn, histology, morphology

## Abstract

**Introduction:**

Terminalia catappa (T. catappa) leaves are used in the treatment of hypertension, diabetes, cough, jaundice, indigestion etc, while the bark is used to treat diarrhea, dysentery, abscesses etc. Due to the acceptance and increased use of medicinal plants in pregnancy, there is a need to evaluate their toxicological profiles in pregnancy for safe use. This study aims to evaluate the toxicological effects of aqueous leaf extract of T. catappa in pregnant rats.

**Methods:**

acute toxicity study was carried out using Organization of Economic Corporation Development (OECD) 423 guidelines. Seventy-five rats (50 females and 25 males) were used at the age of 2 weeks just after weaning. The male rats were separated from the female rats in different cages and allowed to mature for 10 weeks. Then the rats were allowed to mate. After mating, 20 female rats with viable spermatozoa counts were selected and divided into 4 groups of 5 rats each (n=5). The control group received distilled water while the treatment groups II, III, and IV were administered with aqueous leaf extract of T. catappa orally at graded doses of 100, 200, and 400 mg/kg respectively for 21 consecutive days. The daily food and water intake, and weight were recorded. On the 22^th^ day, the rats were anesthetized by chloroform inhalation, and blood samples were collected for haematological and biochemical analysis. The maternal livers, kidneys, and hearts were collected and weighed, and histological studies were carried out. The fetuses were removed and examined. An isolated tissue experiment was carried out on the myometrium of the pregnant rat uterus. The isometric reading of the uterine contractions was recorded.

**Results:**

the oral LD_50_ was found to be ≤2000-5000 mg/kg. There was a significant (p<0.05) increase in the mean food intake at the 100, 200, and 400 mg/kg doses of the extract used on the 14^th^ and 21^st^ days when compared to the 7^th^ day. The renal function test showed a significant (p<0.05) increase for chloride. The liver function test revealed that the extract at 100 mg/kg dose, significantly (p<0.05) increased only Alkaline Phosphatase (ALP) liver enzyme, while at 200 mg/kg dose, only AST liver enzyme was significantly (p<0.05) increased, while at 400 mg/kg dose, ALT, AST, ALP, and albumin increased significantly (p<0.05) when compared to the control group. There was a significant (p<0.05) decrease in the relative organ weight of the liver at 400 mg/kg dose of the extract. The liver histology showed moderate hepatic vacuolation and necrosis, while the histology of the kidney showed slight tubular necrosis.

**Conclusion:**

this study has shown that the aqueous leaf extract of T. catappa is relatively not safe in the pregnant rats. Although it is non-toxic to the fetus, it exhibited tocolytic effect by inhibiting uterine contractions, thus it should be used with caution during pregnancy, especially in the third trimester or during labor.

## Introduction

The use of medicinal plants among pregnant women is a common practice in diverse countries. Several authors have described potential adverse effects arising from the use of medicinal plants in pregnancy such as maternal toxicity, teratogenic, mutagenic, embryotoxic, and abortifacient effects [1]. Cultural and social influences, beliefs about safety, perceived efficacy, and general ease of access are among important factors associated with the use of traditional medicines [2], but long-term clinical experience with little or no scientific data on the efficacy and safety of medicinal plants highlights the rationale for their utilization [3]. Although only 1% of fetal malformations result from exposure to medicinal plants during pregnancy, the number is however significant [4]. Toxicology is the study of adverse effects of chemical, physical or biological agents on living organisms and the ecosystem including the prevention and amelioration of such adverse effects [5], while toxicity can be defined as the relative ability of a substance to cause adverse effects in living organisms [6]. Due to the acceptance and increased use of medicinal plants in pregnancy, there is a need for evaluating the toxicity profile in pregnancy for safety use. The prevalence of congenital fetal abnormalities in women who took herbal medicines is higher but not statistically significant, than that in the women who took pharmaceutical products [7].

Women are the primary customers of herbal medicines and usually use them even during pregnancy. The prevalence of herbal medicine use during pregnancy ranges from 7% to 55% depending on the consumer's geographic location, ethnicity, cultural and social status; Australia 34%, Europe 50%, and USA 9% [7] and 94% in Nigeria [8].

*T. catappa* is a deciduous tree found mainly in subtropical and tropical climates. It belongs to the family *Combretaceae* [9]. It is widely planted throughout the tropical regions of Asia, Africa, and Australia [10]. The juice of its fresh leaves is used for the treatment of cough, jaundice, indigestion, and diabetes while the bark is used to treat dysentery, diarrhoea, and abscesses [11]. Pharmacological investigations carried out on the plant revealed that the bark has anti-diarrhoea activity [12], while the leaves have antioxidant activity [13], anti-inflammatory activity [14], anti-diabetic activity [15], and antimicrobial activity [16]. Considering the prevalent use of herbal medicines, and the ethnomedicinal importance of *T. catappa*, it will be of paramount importance to evaluate the toxicological consequences of sub-acute oral administration of aqueous leaf extract of *T. catappa* in pregnant rats, to establish its safety profiles in pregnancy.

## Methods

**Study design:** it is an experimental study aiming to evaluate the toxicological profiles of the aqueous leaf extract of *Terminalia catappa* in pregnant rats.

**Animal study:** fifty (50) female virgin rats and twenty-five (25) male rats at the age of two weeks (just after weaning) were procured from the animal house, Department of Pharmacology and Therapeutics, Ahmadu Bello University, Zaria. The animals were separated based on their sex in different cages (5 rats per cage) in a well-ventilated condition at ambient temperature and fed with standard animal feeds with adequate access to water *ad-libitum*. The animals were allowed to attain ten (10) weeks of maturity as earlier described by [17] in the Pharmacology Department, Bayero University Kano.

Fresh leaves of *T. catappa* were collected in the month of June, 2018 at Bayero University Kano, Kano State-Nigeria. The plant samples were identified and authenticated by a botanist at the herbarium unit of the Biology Department, Bayero University Kano. The plant samples were compared with an already deposited specimen and the voucher number (BUKHAN 0389) was given for reference. The leaves were shade-dried to a constant weight and size reduced to a fine powder. The powdered leaves weighing 1000 g were macerated in 4 L of distilled water for 48 hours. The marcs were removed and the liquid was filtered with filter paper (Whatman grade 1) [13]. The filtrate was then freeze- dried [18]. The extract was collected, weighed, and stored in an airtight container and placed in a desiccator until use. The percentage yield of the extract was 8.4%.

**Acute toxicity studies:** median lethal dose (LD_50_) of the aqueous leaf extract of *T. catappa* was determined using the OECD 423 guideline [19]. Six male rats were fasted overnight prior to dosing. Following the period of fasting, the rats were weighed and a 2000 mg/kg dose of the extract was administered. Food was then withheld for three (3) hours. The animals were closely observed for 24 hours and thereafter for 14 days for signs of toxicity and mortality [19].

**Mating of the rats:** vaginal smear tests were carried out on the female rats prior to mating to know the phase of the estrous cycle before introducing the male rats [20]. Two female rats were kept together with one male rat (2: 1) for 24 hours. The male rats were removed thereafter. Mating was confirmed by the presence of spermatozoa in the vagina by the swabbing method of vaginal smear examination (microscopic observation) as described by [21]. Twenty female rats (160 - 200 g) with viable spermatozoa deposits were selected and that day was taken as day 1 of pregnancy [22].

**Prenatal developmental toxicity study:** the twenty selected rats (160 - 200 g) were divided into four groups of five rats each (n=5) with a slight amendment to OECD 414. Group I received distilled water and served as control, while groups II, III, and IV were administered orally with the graded concentrations of the extract at doses of 100, 200, and 400 mg/kg respectively with the aid of an oral gavage needle for 21 consecutive days. The weight changes, food intake, and water intake were recorded daily for 21 consecutive days.

**Collection of blood samples:** the female rats were anesthetized by chloroform inhalation [23] and blood samples were collected from the ophthalmic venous plexus using a micro-capillary pipette [24].

**Determination of hematological parameters:** the red blood cells (RBC), white blood cells (WBC) heamoglobin (Hb), packed cell volume (PCV), mean corpuscular volume (MCV), mean corpuscular haemoglobin (MCH), and mean corpuscular haemoglobin concentration (MCHC) were determined using Rayto hematological analyzer (RT-7600).

### Determination of biochemical parameters

**Renal function test:** blood urea, creatinine, sodium, potassium, bicarbonate, and chloride were measured.

**Liver function test:** Serum Aspartate Aminotransferase (AST), Serum Alanine Aminotransferase (ALT), Serum Alkaline Phosphatase (ALP), total protein, albumin and bilirubin levels were measured. These were carried out at Anatomy Department, Ahmadu Bello University Zaria, Kaduna, Nigeria.

**Termination of pregnancy:** the fetuses were removed by laparotomy and examined on the 22^nd^ day. The maternal livers, kidneys, and hearts were collected and weighed as described by [25]. Histology of the livers and kidneys was carried out using standard protocols.

**Fetal studies:** the fetal number, fetal weight, crown-rump length, tail length, structural malformation, and live and dead fetuses were recorded [26]. The fetuses were immediately fixed in 10% formal saline (UK Chemical Suppliers) for 5 days to enable a clearer vision of the fetal morphology.

**Histopathology method:** the histology of the kidney and liver was carried out at the Anatomy Department, Ahmadu Bello University Zaria, Kaduna State, Nigeria. Histology of the liver and kidney were considered due to limited resources. The liver and kidney were harvested from the animal and immediately fixed in 10% formal saline for 48 hours. After the fixation process was completed, the tissues were undergone tissue processing routine processes by passing through ascending grades of methanol from 70% to 90% and 100% for a period of 12 hours to properly dehydrate them after which they were cleared in xylene for 2 hours and then infiltrated and embedded in liquid paraffin wax. The tissues were then cut using rotary microtome at 5 um thickness and the section were stained using haematoxylin and eosin staining technique [27].

**Isolated tissue experiment:** composition of De-jalon´s soloution: NaCl (LOBA Chemie Lab. Reagents and Fine Chemicals)…….. 90g; KCl (Guangdong Guanghua Sci-Tech. Co. China)………...........4.2g; D-Gluose (Park Scientific Ltd)……………………………….....5g; NaHCO3 (BDH Laboratories England)………………………...5g; CaCl2 1M (BDH Laboratories England)…………….…………..2.7ml; aerating gas: 95% O2 + 5% CO2 (aquarium air pump; Model SB-108); distilled water………………………………………………10L.

**Preparation of isolated pregnant uterus:** according to the method of [28], the pregnant rats were sacrificed and a 3 cm length uterine muscle was removed, fatty deposits on the uterus were gently removed with forceps and immersed in De-jalon´s solution. The uterus was placed in an organ bath (ORCHID Scientific; Model No. OBID) containing 25 mL De-jalon´s solution, bubbled with atmospheric air. The temperature of the organ bath was maintained at 37 ± 1 °C. One end of the preparation was attached to a stainless stall rod and the other was attached to a force transducer which was placed under optimum resting tension for 30 minutes. The isometric reading of the uterine contractions was recorded by the oscillograph (Harvard App. Ltd).

**Effect of contractile agonist on the uterus:** oxytocin (Rotex: Batch no.VN-9978-10) was added to the tissue in the bath in a cumulative manner to achieve organ bath concentrations (0.007 - 0.053 μg/mL). After each addition of the agonist the tissue was properly washed with De-jalon´s solution. The isometric reading of the uterine contractions was recorded by the oscillograph. Also Misoprostol (Pfizer: Batch no. B16951) was added to the tissue in the bath in a cumulative manner to achieve organ bath concentrations (0.008 - 0.064 μg/mL). After each addition of the agonist, the tissue was properly washed. The isometric reading of the uterine contractions was recorded by the oscillograph.

**Effect of the extract on the uterus:** extract 10 mg/mL stock solution was selected for the study. This was added to the tissue in the bath in a cumulative manner to achieve organ bath concentrations (40 - 320 μg/mL). After each addition of the extract the tissue was properly washed with De-jalon´s solution. The isometric reading of the uterine contractions was recorded by the oscillograph.

**Interactive studies of extract and oxytocin:** the extract was added to the tissue in the bath in a cumulative manner to achieve organ bath concentrations (40 - 320 μg/mL) and a constant concentration of oxytocin (0.03 μg/mL) was added after one minute. The tissue was washed with De-jalon´s solution after each interaction. The response was recorded on the oscillograph.

**Interactive studies of extract and misoprostol:** the extract was added to the tissue in the bath in a cumulative manner to achieve organ bath concentrations (40 - 320 μg/mL) and a constant concentration of misoprostol (0.03 μg/mL) was added after 1 minute. The tissue was washed with De-jalon´s solution after each interaction. The response was recorded on the oscillograph.

**Ethical approval:** the Ethical Committee, College of Health Sciences Bayero University Kano, Nigeria provided clearance for the study (BUK/CHS/HREC/VII/66).

**Statistical methods:** data obtained were statistically analyzed using SPSS (Version 20) and expressed as mean ± standard error of the mean (S.E.M). Differences between means were analyzed using paired-sample t-test, one-way analysis of variance (ANOVA), and repeated measure ANOVA followed by Bonferroni post hoc test as the case may be p< 0.05 were considered significant.

## Results

**Medium lethal dose (LD_50_) determination:** the oral median lethal dose (LD50) in rats was found to be greater than 2000 mg/kg body weight using the OECD method thus category 5 of chemical classification of the OECD guideline 423 (19). There were no changes in eyes, mucous membranes, and behavioral patterns. There was no sign of tremor, convulsion, salivation, diarrhea, sleep, and/or death.

**Effect of the extract on body weight of pregnant rats over 21 consecutive days administration:** the extract did not cause any significant (p<0.05) difference in the mean body weight gain of the rats at 200 mg/kg and 400 mg/kg doses compared to the mean body weight gain of the control group. Also, there was no significant (p<0.05) difference in the mean body weight gain at day 14 and day 21 when compared to day 7 over time (Table 1).

**Table 1:** effect of the extract on body weight of pregnant rats over 21 consecutive days administration

Treatment (mg/kg)	Mean weight gain (g)		
	Day-7	Day-14	Day-21
Distilled water (1 mL/kg)	16.20 ± 2.75	15.20 ± 3.81	17.20 ± 2.58
Extract (100)	18.60 ± 2.98	15.60 ± 1.50	11.40 ± 1.50
Extract (200)	12.00 ± 1.38	17.00 ± 2.17	15.00 ± 2.14
Extract (400)	11.00 ± 2.53	11.00 ± 2.70	12.60 ± 1.75

Values expressed as mean ± S.E.M; n=5; *=p<0.05; S.E.M: standard error of the mean

**Effect of the extract on mean water intake in pregnant rats over 21 consecutive days administration:** the extract did not cause any statistically significant (p<0.05) difference in the mean daily water intake at 100, 200 and 400 mg/kg doses compared to the control group (Table 2).

**Table 2:** effect of the extract on mean water and food intake in pregnant rats over 21 consecutive days administration

Treatment (mg/kg)	Mean water intake (mL)			Mean food intake (g)		
	Day-7	Day-14	Day-21	Day-7	Day-14	Day-21
Distilled water (1 mL/kg)	156.00 ± 9.45	163.57 ± 7.38 (4.85%)	175.71 ± 6.76 (12.63%)	206.14 ± 9.76	248.57 ± 9.78* (20.58%)	286.14 ± 9.12* (38.81%)
Extract (100)	169.29 ± 11.20	176.71 ± 4.26 (4.38%)	175.29 ± 3.39 (3.54%)	211.85 ± 9.51	239.85 ± 6.65* (13.22%)	265.00 ± 9.37* (25.09%)
Extract (200)	160.00 ± 11.20	161.86 ± 13.74 (1.16%)	161.00 ± 8.98 (0.63%)	235.29 ± 8.10	289.42 ± 5.99* (23.01%)	291.57 ± 5.57* (23.92%)
Extract (400)	159.29 ± 9.79	179.29 ± 7.67 (12.56%)	171.00 ± 4.00 (7.35%)	233.71 ± 8.29	290.00 ± 5.89* (24.09%)	300.00 ± 0.01* (28.36%)

Values expressed as mean ± S.E.M.; n=5; *=p<0.05; S.E.M.: standard error of the mean

**Effect of the extract on mean food intake in pregnant rats over 21 consecutive days administration:** the extract caused a statistically significant (p<0.05) increase in the mean food intake at 100, 200, and 400 mg/kg doses used on the 14^th^ and 21^st^ days when compared to 7^th^day (Table 2).

**Effect of the extract on hematological parameters of pregnant rats:** the extract caused a statistically significant (p<0.05) increase in the value of the granulocytes at 100 mg/kg dose compared to the control group. However, there was increase in the other haematological parameters but were not statistically significant (p<0.05) (Table 3).

**Table 3:** effect of the extract on hematological parameters of pregnant rats

Haematological parameters	Treatment (mg/kg)			
	Distilled water (1 mL/kg)	Extract (100)	Extract (200)	Extract (400)
WBC (10^3/u L)	4.36 ± 0.51	5.74 ± 0.50	3.86 ± 0.26	5.32 ± 0.53
LYMPH (10^3/u L)	3.82 ± 0.47	4.84 ± 0.28	5.16 ± 0.51	4.26 ± 0.52
GRAN (10^3/u L)	2.40 ± 0.34	3.72 ± 0.19*	2.74 ± 0.49	3.04 ± 0.29
RBC (10^6/u L)	4.48 ± 0.25	4.88 ± 0.08	4.64 ± 0.18	4.68 ± 0.24
Hb (g/dL)	12.82 ± 0.79	13.76 ± 0.46	13.30 ± 0.68	13.38 ± 0.68
HCT (%)	38.74 ± 2.46	40.62 ± 2.03	39.78 ± 2.11	40.14 ± 2.17
MCH (pg)	30.54 ± 1.36	30.52 ± 0.66	31.64 ± 0.60	30.16 ± 1.32
MCHC (g/dL)	32.42 ± 1.13	32.50 ± 1.08	31.06 ± 0.74	33.60 ± 1.14

Values expressed as Mean ± S.E.M, n=5, *=p<0.05. WBC: white blood cells; LYMPH: lymphocyte; GRAN: granulocytes; RBC: red blood cells; Hb: heamoglobin; HCT: heamatocrite; MCH: mean corpuscular haemoglobin; MCHC: mean corpuscular haemoglobin concentration; S.E.M: standard error of the mean

**Effect of the extract on renal function parameters in pregnant rats:** there was an increase in the value of urea as the doses of the extract increased compared to the control group. Conversely, there was also a decrease in the values of sodium as the doses of the extract increased. However, at the dose of 400 mg/kg there was a statistically significant (p<0.05) increase in the values of chloride compared to the control group (Table 4).

**Table 4:** effect of the extractt on renal function parameters in pregnant rats

Treatment (mg/kg)	Urea (mg/dL)	Sodium (mmol/L)	Potassium (mmol/L)	Creatinine (meq/L)	Chloride (mg/dL)	Hydrogen bicarbonate (mg/dL)
Distilled water (1 mL/kg)	63.90 ± 9.12	128.46 ± 4.92	20.42 ± 0.80	0.92 ± 0.67	25.00 ± 0.71	97.00 ± 4.44
Extract (100)	73.38 ± 5.67	120.30 ± 5.21	18.20 ± 0.38	1.06 ± 0.12	27.60 ± 1.50	85.20 ± 2.87
Extract (200)	74.22 ± 11.03	115.12 ± 3.12	22.44 ± 1.15	0.90 ± 0.07	29.80 ± 2.60	82.80 ± 2.33
Extract (400)	82.68 ± 5.92	115.44 ± 3.68	21.12 ± 1.98	0.92 ± 0.11	34.60 ± 2.46*	86.00 ± 5.83


Values expressed as mean ± S.E.M; n=5; *=p<0.05; S.E.M.: standard error of the mean

**Effect of the extract on liver function parameters in pregnant rats:** the extract at 100 mg/kg dose, significantly (p<0.05) increased only ALP liver enzyme, while at 200 mg/kg, only AST liver enzyme was significantly (p<0.05) increased, while at 400 mg/kg, ALT, AST, ALP, and albumin increased significantly (p<0.05) when compared to the control group (Table 5).

**Table 5:** effect of the extract on liver function parameters in pregnant rats

Treatment (mg/kg)	ALT (IU/L)	AST (IU/L)	ALP (IU/L)	Total protein (g/dL)	Albumin (g/dL)	Total bilirubin (mg/dL)
Distilled water (1 mL/kg)	23.60 ± 2.34	213.80 ± 7.25	27.46 ± 1.14	13.14 ± 0.97	3.16 ± 0.19	0.68 ± 0.19
Extract (100)	31.80 ± 4.81	277.00 ± 9.73	41.96 ± 3.24*	10.50 ± 0.75	3.06 ± 0.23	0.80 ± 0.03
Extract (200)	39.20 ± 3.22	291.00 ± 3.89*	34.18 ± 2.73	11.28 ± 1.00	3.38 ± 0.14	0.70 ± 0.03
Extract (400)	67.00 ± 8.12*	290.60 ± 3.64*	37.28 ± 2.87*	7.10 ± 0.69	4.36 ± 0.42*	0.93 ± 0.01

Values expressed as mean ± S.E.M.; *p< 0.05; n=5; AST: aspartate aminotransferase; ALT: alanine aminotransferase; ALP: alkaline phosphatase; S.E.M.: standard error of the mean

**Effect of the extract on the relative organ-body weight of pregnant rats:** the extract caused a significant (p<0.05) decrease in the relative weight of the kidney and liver at 400 mg/kg dose compared to the control group. There was also a significant (p<0.05) increase in the relative weight of the heart at 100 mg/kg dose compared to the control group (Table 6).

**Table 6:** effect of the extract on relative organ-body weight of pregnant rats

Treatment (mg/kg)	Relative organ weight (%)		
	Heart weight	Kidney weight	Liver weight
Distilled water (1 mL/kg)	0.51 ± 0.03	3.60 ± 0.32	0.34 ± 0.02
Extract (100)	0.63 ± 0.04*	4.13 ± 0.36	0.39 ± 0.02
Extract (200)	0.48 ± 0.02	3.02 ± 0.20	0.35 ± 0.02
Extract (400)	0.48 ± 0.01	2.54 ± 0.10*	0.27 ± 0.01*


Values expressed as mean ± S.E.M; n=5; *=p<0.05; S.E.M.: standard error of the mean

**Effect of the extract on fetal parameters:** the extract did not cause any significant (p<0.05) change in the fetal weight, crown-rump length, tail length, and number of fetuses at 100, 200, and 400 mg/kg doses compared to the control group (Table 7).

**Table 7:** effect of the extract on fetal parameters

Treatment (mg/kg)	Fetal weight (g)	Crown-rump length (cm)	Tail length (cm)	No of fetuses
Distilled water (1 mL/kg)	4.57 ± 0.09	4.26 ± 0.08	1.29 ± 0.03	5.80 ± 0.97
Extract (100)	4.38 ± 0.13	4.07 ± 0.10	1.28 ± 0.02	4.40 ± 1.08
Extract (200)	4.75 ± 0.11	4.11 ± 0.07	1.32 ± 0.01	5.20 ± 0.66
Extract (400)	4.47 ± 0.08	4.05 ± 0.09	1.29 ± 0.02	6.60 ± 1.63

Values expressed as mean ± S.E.M; n=5; *=p<0.05; S.E.M.: standard error of the mean

**Figure 1:**
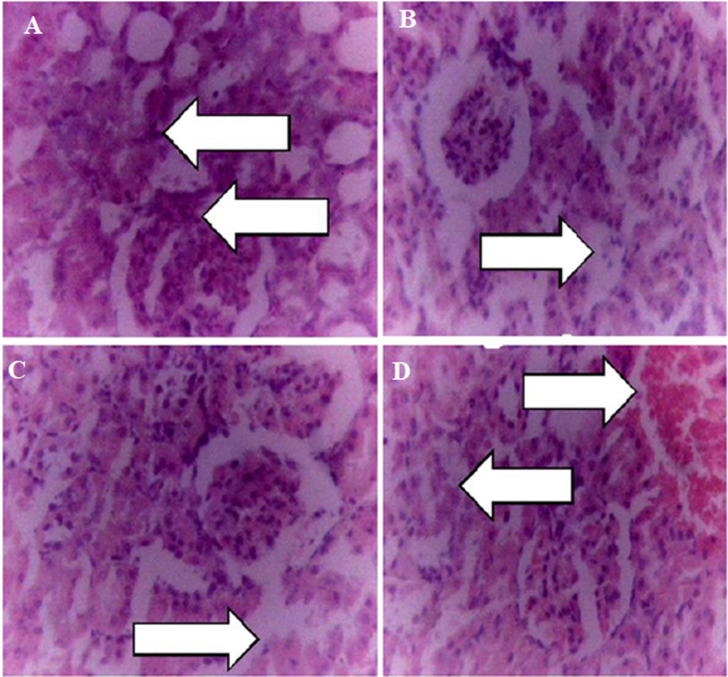
A, B, C, D) effect of the extract on histology of the kidney

**Effect of the extract on histology of the kidney:** the kidney showed normal tubules and glomerulus in the control group (Figure 1A). At doses of 100 and 200 mg/kg the kidney showed slight tubular necrosis (Figure 1(B,C)) while at dose of 400 mg/kg the kidney showed slight tubular necrosis and hemorrhage (Figure 1D).

**Figure 2:**
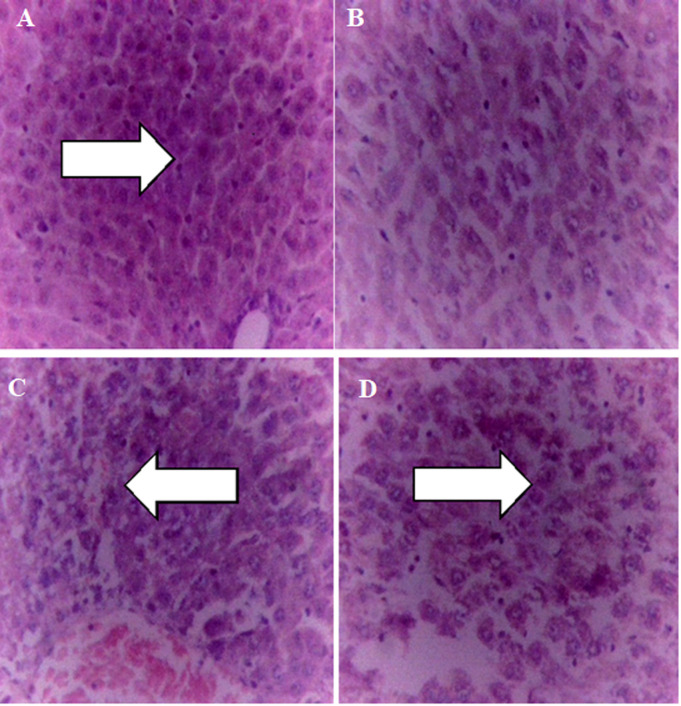
A, B, C, D) effect of the extract on histology of the liver

**Effect of the extract on histology of the liver:** the liver showed normal hepatocytes in the control group (Figure 2A) and at the dose of 100 mg/kg (Figure 2B). At the dose of 200 mg/kg, the liver showed moderate hepatic vacuolation and necrosis (Figure 2C) while at 400 mg/kg dose the liver showed moderate hepatic necrosis (Figure 2D).

**Figure 3:**
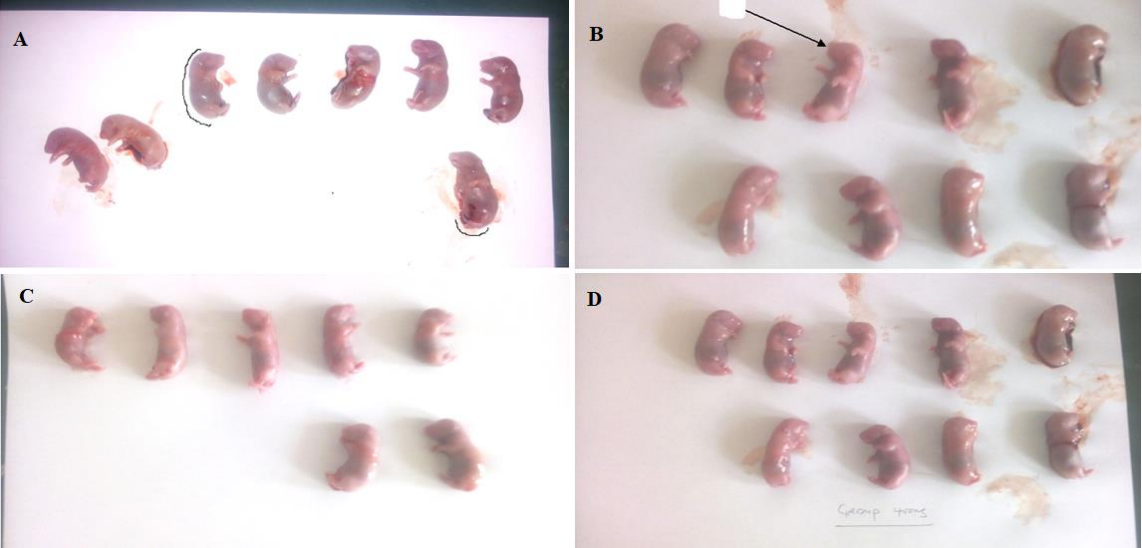
A, B, C, D) effect of the extract on fetal morphology

**Effect of the extract on fetal morphology:** at the doses used, there was no structural malformation of the crown-rump length, tail length, head, limbs, or palate of the fetuses, and no fetal death recorded (Figure 3 (A,B,C,D).

**Figure 4:**
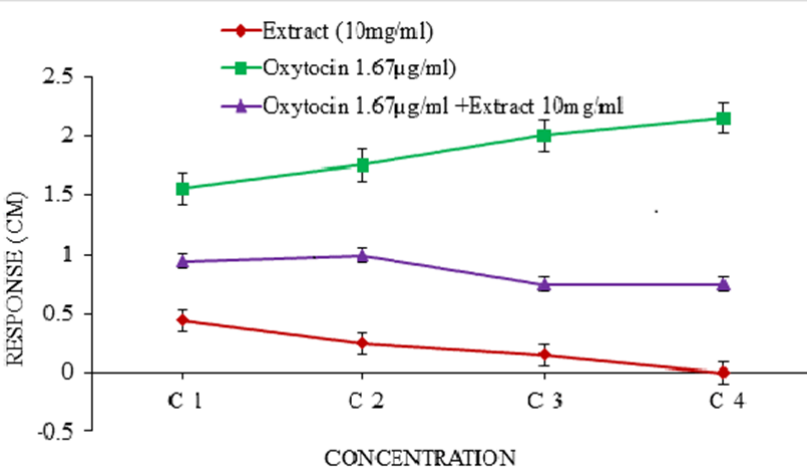
effect of the extract on isolated pregnant rat uterus in the presence and absence of oxytocin

**Effect of the extract on isolated pregnant rat uterus in the presence and absence of oxytocin:** the extract inhibited the normal contraction of the uterus in a dose-dependent manner. On the other hand, oxytocin stimulated uterine contraction in a dose-dependent manner. However, the contraction induced by oxytocin decreased in the presence of the extract and the curve of oxytocin shifted to the left (Figure 4).

**Figure 5:**
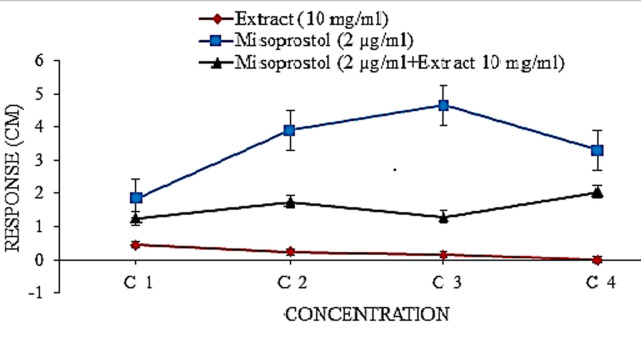
effect of the extract on isolated pregnant rat uterus in the presence and absence of misoprostol

**Effect of the extract on isolated pregnant rat uterus in the presence and absence of misoprostol:** the extract inhibited the normal contraction of the uterus in a dose-dependent manner while misoprostol stimulated uterine contraction. However, the contraction induced by misoprostol decreased in the presence of the extract and the curve of misoprostol shifted to the left (Figure 5).

## Discussion

The result obtained from the acute toxicity study showed a reasonable safety profile of the aqueous leaf extract of *T. catappa* at the dose of 2000 mg/kg. The oral LD *50* of the extract was estimated to be ≤ 2000 - 5000 mg/kg according to the OECD 421 guideline for the classification of chemicals. This suggests that the phytochemical constituents of the extract are relatively safe [19], and this is in line with the work of [29] and [30]. This may be responsible for its widespread use in different ethnomedicines [29]. Although, [31] found that the unripe fruits of *T. catappa* is toxic to cattle and sheep when eaten, causing kidney necrosis.

Changes in body weight, food, and water intake are signs used to detect health status in animals [32]. An adequate food and water intake helps to maintain the balance between total energy intake and daily energy expenditure. Conversely, inadequate water intake relative to recommendation contributes to a decline in physical activities [33]. Change in mean body weight (Table 1) of the rats may be as a result of pregnancy, number of fetuses, metabolic factors, and biochemical factors [34]. The observed increase in the mean food intake (Table 2) may also be due to either the extract or other related factors such as hormonal changes and pregnancy. Estrogen and progesterone interfere with food and water intake, energy balance, and fluid retention [35].

In consideration of the effect of the extract on hematological parameters (Table 3), the white blood cell and lymphocyte counts showed a marginal increase but were not statistically significant (p<0.05). This result agrees with the work of [36] who reported that there was no significant (p<0.05) change in the white blood cell and lymphocyte counts in albino wistar rats given with *T. catappa* leaves decoction. An increase in the number of white blood cells usually indicates an increase in physical or emotional stress, infections, and an immune system-associated health conditions that may increase white blood cell production [37]. The result also revealed that there was a significant increase (p<0.05) in the number of granulocyte which is an indication of inflammatory processes or kidney diseases [37].

Serum biochemical tests are frequently and widely used to diagnose kidney, liver, and heart-associated health conditions amongst others, and also in monitoring the response of the body when exposed to exogenous toxins [38]. Renal function test (Table 4) showed that chloride ion (Cl-) was significantly (p<0.05) increased at the 400 mg/kg dose. The observed increase in chloride ion (Cl-) is an indication of decreased renal perfusion associated with renal tubular necrosis [39]. In addition to that, there was a slight increase in the mean value of creatinine and urea but not statistically significant (p<0.05). Increase in the blood levels of creatinine and urea is the hallmark of severe infections and kidney failure [40]. At 200 and 400 mg/kg doses of the extract, the histology of the kidney (Figure 1) revealed slight to moderate tubular necrosis and hemorrhage. Acute tubular necrosis is often caused by a lack of blood flow and oxygen supply to the kidney, hemorrhage, and renal damage by toxicants and harmful substance [41].

The liver is one of the most metabolically active organs in the body. Liver enzymes help in the maintenance of the integrity and architecture of the liver. In consideration of the experimental results (Table 5), the observed increase in bilirubin may be an indication of liver injury and heamolysis. The levels of ALT, AST, ALP, and albumin in this study were significantly (p<0.05) increased at the 400 mg/kg dose. The histology of the liver (Figure 2) also revealed that at 200 and 400 mg/kg doses, the extract induced slight vacuolation and moderate hepatic necrosis. The increase in ALT, AST, and ALP may be due to the leakage of these enzymes from the liver cytosol into the blood-stream which is an indication of liver injury or muscle damage [42].

Change in organ weight is a sensitive indicator of chemically induced changes to organs, and organ weight is an index of swelling, atrophy or hypertrophy [43]. In consideration of the effect of the extract on the relative organ weight of the rats (Table 6), the observed decrease in liver and kidney weight generally reflects a loss in functional mass associated with atrophy [44]. This is in support of the result obtained in the renal function test (Table 4) and liver function test (Table 5). However, this is contrary to the report of [45] that the leaf extract of *T. catappa* possessed hepatoprotective and antioxidant properties through suppression of oxidative stress and apoptosis. This may be due to differences in the doses used for the experiment and the geographical source of the plant samples.

The fetal weight, crown-rump length, and tail length are used to evaluate fetal toxicity, and fetal weight is strongly associated with perinatal morbidity and mortality [46]. Crown-rump length is use to estimate gestational period [46]. The fetal parameters (Table 7) showed that the extract has no effect on the crown-rump length, fetal weight, tail length as well as the fetal numbers. Moreover, physical examination of the external morphology of the fetuses (Figure 3) revealed that there was no structural malformation of the head, limbs, palate, tail, and general body structure. This inferred that the aqueous leaf extract of *T. catappa* may be non-toxic to the fetuses.

Under suitable physiological conditions, isolated rat uterus are capable of exhibiting spontaneous rhythmic contractions and are able to respond to prostaglandin and oxytocic agents in a similar fashion to their *in vivo* activity [47]. The aqueous leaf extract of *T. catappa* neither induced contraction nor relaxation of the uterus but inhibited the spontaneous rhythmic contraction of the uterus. This implies that the extract has affinity but no efficacy; this is a characteristic of an antagonist. In the presence of the aqueous leaf extract of *T. catappa*, oxytocin-induced contractions were significantly (p<0.05) inhibited thereby shifting the curve of oxytocin to the right (Figure 4). Similarly, misoprostol-induced contractions were also significantly (p<0.05) inhibited and its curve shifted to the right (Figure 5). On the other hand, it is also possible the extract acts probably by inhibiting the hydrolytic pathway and subsequently inhibiting the release of intracellular calcium (Ca^2+^) which plays a significant role during uterine contraction. This suggests that the extract exhibited tocolytic effect by inhibiting uterine contractions induced by oxytocin and misoprostol and this constitutes the basis of drugs used in tocolysis [48].

## Conclusion

In this study, the results revealed that the aqueous leaf extract of *T. catappa* is relatively not very safe in pregnant rats, though it has been shown to be non-toxic to the fetuses. Additionally, the extract exhibited tocolytic effect by inhibiting uterine contractions, thus it should be used with caution during pregnancy, especially in the third trimester or during labor. Furthermore, our study might be considered as the first report on the tocolytic properties of *T. catappa*.

### What is known about this topic

- *Several potential adverse effects arise from the use of medicinal plants in pregnancy, such adverse effects include maternal toxicity, teratogenic, mutagenic, embryotoxic, and abortifacient;*
- *Traditionally, the juice of the fresh leaves of T. catappa has been used for the treatment of cough, jaundice, indigestion, diabetes mellitus etc., while the bark is used to treat dysentery, diarrhea, abscesses etc.;*
- *Additionally, pharmacological investigations revealed that the bark of the plant has anti-diarrheal activity, while the leaves have antioxidant, anti-inflammatory, anti-diabetic, and antimicrobial activities*.

### What this study adds

- *This study has evaluated the toxicological effects of aqueous leaf extract of T. catappa on pregnant rats and the fetuses;*
- *It was found that the plant exhibited a tocolytic effect by inhibiting uterine contractions;*
- *In the sub-chronic studies, there were mild to moderate toxicities to the liver (hepatic vacuolation and necrosis) and kidney (tubular necrosis), though it has shown to be non-toxic to the fetuses*.
